# Cracking the code: high ferritin load with Salmon-Colored skin episodes

**DOI:** 10.1093/omcr/omae092

**Published:** 2024-08-23

**Authors:** Paraash Satyal, Walia Sukhcharan, Neriy Yakubov, Benson Babu

**Affiliations:** Department of Hospital Medicine, Internal Medicine Wyckoff Medical Center, 374 Stockholm Street, Brooklyn, New York, 11237, United States; Department of Hospital Medicine, Internal Medicine Wyckoff Medical Center, 374 Stockholm Street, Brooklyn, New York, 11237, United States; Department of Hospital Medicine, Internal Medicine Wyckoff Medical Center, 374 Stockholm Street, Brooklyn, New York, 11237, United States; Department of Hospital Medicine, Internal Medicine Wyckoff Medical Center, 374 Stockholm Street, Brooklyn, New York, 11237, United States; Assistant Professor New York Medical College, 40 Sunshine Cottage Rd, Valhalla, NY 10595, United States

**Keywords:** adult-onset still’s disease, salmon-colored rash, ferritin, artificial intelligence

## Abstract

A 37-year-old previously healthy male presented to the Emergency Department with a two-week history of intermittent fevers, joint pain, sore throat, and a diffuse salmon-colored rash. Examination revealed a pruritic rash with joint swelling and red spots in the oropharynx. Initial sepsis management was instituted, but subsequent investigations, including infectious, hematologic, and autoimmune workups, were inconclusive. Notably, elevated ferritin levels prompted consideration of life-threatening conditions like Hemophagocytic Lymphohistiocytosis, which was ultimately ruled out. Adult-onset Still’s Disease (AOSD) emerged as the leading diagnosis following the exclusion of other potential causes. A skin biopsy was performed with non-specific findings and corticosteroid treatment led to significant improvement. This case illustrates the clinical decision-making process of diagnosing AOSD and highlights the potential utility of novel AI technology in dermatologic assessments.

## Case presentation

A 37-year-old man with no medical history presented to the Emergency Department (ED) with a two-week history of intermittent fevers, joint pain, sore throat, and a diffuse salmon-colored rash. The pruritic rash started on his abdomen and spread to his arms, back, and legs. The joint pains were intermittent but localized to bilateral knees and elbows. His history revealed no recent travel, sexual partners, or medication changes.

Upon examination, he had a diffuse rash with flat and raised lesions all over his skin, sparing the face, palms, and soles. Oral examination showed red spots in the oropharynx, with symmetric joint swelling, and no other notable findings on respiratory, cardiovascular, neurologic, or abdominal examination.

In the ED, the patient presented with sepsis criteria, prompting initial treatment with IV Zosyn and Vancomycin. However, subsequent tests yielded no clear infectious source. Noteworthy findings included elevated ESR, CRP, liver function tests (AST 460, ALT 847), and extremely high ferritin levels (peaking at 92999) [1].

## Approach to diagnosis

Elevated ferritin levels and a maculopapular rash narrowed down multiple possible diagnoses. Life-threatening conditions such as Hemophagocytic Lymphohistiocytosis (HLH) were considered; however, the likelihood was low due to the absence of typical symptoms such as neurologic symptoms, multi-organ involvement, lymphadenopathy, hepatosplenomegaly, cytopenia, and hypofibrinogenemia [2].

## Infectious workup

Infections can raise ferritin levels as part of the acute phase response. Extensive testing ruled out various infectious diseases, all returning negative results.

1. HBsAg, HB core IgG, HB core IgM, HBV, HCV, and HIV DNA.
2. EBV IgG, IgM, CMV IgG, IgM, Toxoplasma IgG, IgM, parvovirus PCR, and Quantiferon- TB Gold.
3. Brucella IgG, IgM, and Bartonella IgG.
4. *Treponema Pallidum* and GC, and Chlamydia Urine Ag.
5. Tick-born disease Ab panel.
6. Chikungunya IgG.
7. Urine Leptospira Ag, DNA.
8. Serological tests for streptococcal infection.
9. Blood cultures, including fungal.

## Diagnostic imaging and hematologic workup

CT chest, abdomen, and pelvis scans showed no lymphadenopathy or hepatosplenomegaly. Serum protein electrophoresis (SPEP) and urine protein electrophoresis (UPEP) were done, and SPEP suggested inflammatory rather than malignant causes, ruling out lymphoma, leukemia, and solid tumors.

## Autoimmune workup

The negative infectious and hematologic tests raised suspicion of acute rheumatic fever (ARF), meeting Jones’ criteria. However, further tests, including echocardiogram, ASO and ADB titers, streptococcal probe, and blood cultures, all returned negative, ruling out ARF. Rheumatoid arthritis was improbable due to age, lack of metacarpophalangeal joint or proximal interphalangeal joint involvement, and negative X-ray. SLE was unlikely based on criteria and ANA titer. Inflammatory bowel disease and sarcoidosis were improbable due to the absence of GI and respiratory symptoms.

## Additional workup

Hemochromatosis, marked by excessive iron absorption and high ferritin levels, and Porphyria cutanea tarda, a metabolic disorder causing heme biosynthesis enzyme deficiency, were considered due to skin discoloration. Both can cause reddish-brown or bronze/salmon-colored rashes.

Diagnosing adult-onset Still’s disease (AOSD) requires thorough evaluation to rule out other conditions. In this case, meeting Yamaguchi criteria, while other infectious, hematologic, and autoimmune diseases were excluded [3]. A skin biopsy was performed for the skin rash, though non-specific, showing perivascular dermatitis. The salmon-colored rash, while a non-specific dermatologic finding, could benefit from novel AI technology using smartphone cameras to help narrow down the differential diagnosis, especially in areas with limited access to specialists [4].

## Treatment

**Figure 1:**
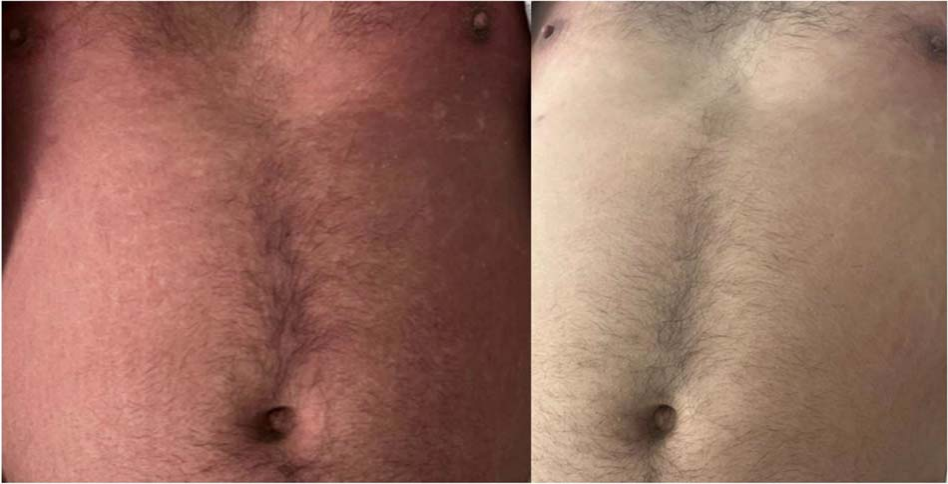
Image on the Left side shows rashes before treatment and image on the right shows clearing of rashes after treatment.

Initially, Naproxen 500 mg PO q12hrs was prescribed for two weeks, but after seven days without improvement, treatment intensified with three days of IV Methylprednisolone 1 g daily with notable improvement, followed by prednisone for a few weeks. Methotrexate will be initiated after prednisone completion. See Fig. 1 for rash progression before and after treatment.

The cornerstone treatment for AOSD is anti-inflammatory drugs like glucocorticoids, which often yield a significant response. However, concerns about adverse effects are growing. While other Disease-Modifying Anti-Rheumatic Drugs have been used, Ilaris (canakinumab) injection is one FDA-approved option. It inhibits IL-1 effects, reducing inflammation in this disorder [5, 6]. Additionally, AI-driven computer vision technology embedded in smart phones has shown promise in aiding dermatologic differential diagnosis, particularly in resource-limited settings, for early detection and intervention [4]. This AI-based technology is currently undergoing testing and validation in the clinical setting to ensure its efficacy and reliability.

## Declaration of generative AI and AI-assisted technologies in the writing process

During the preparation of this work, the author(s) used Grammarly and Notion AI to edit the manuscript. After using this tool/service, the author(s) reviewed and edited the content as needed and took (s) full responsibility for the content of the publication.
